# Supplementary material for: The Influence of New Silicate Cement Mineral Trioxide Aggregate (MTA Repair HP) on Metalloproteinase MMP-2 and MMP-9 Expression in Cultured THP-1 Macrophages
Source: Int J Mol Sci. 2020 Dec 30;22(1):295. doi: 10.3390/ijms22010295 (PMC7795909; doi:10.3390/ijms22010295)
Supplement: Supplementary file 1 [file ijms-22-00295-s001.pdf]

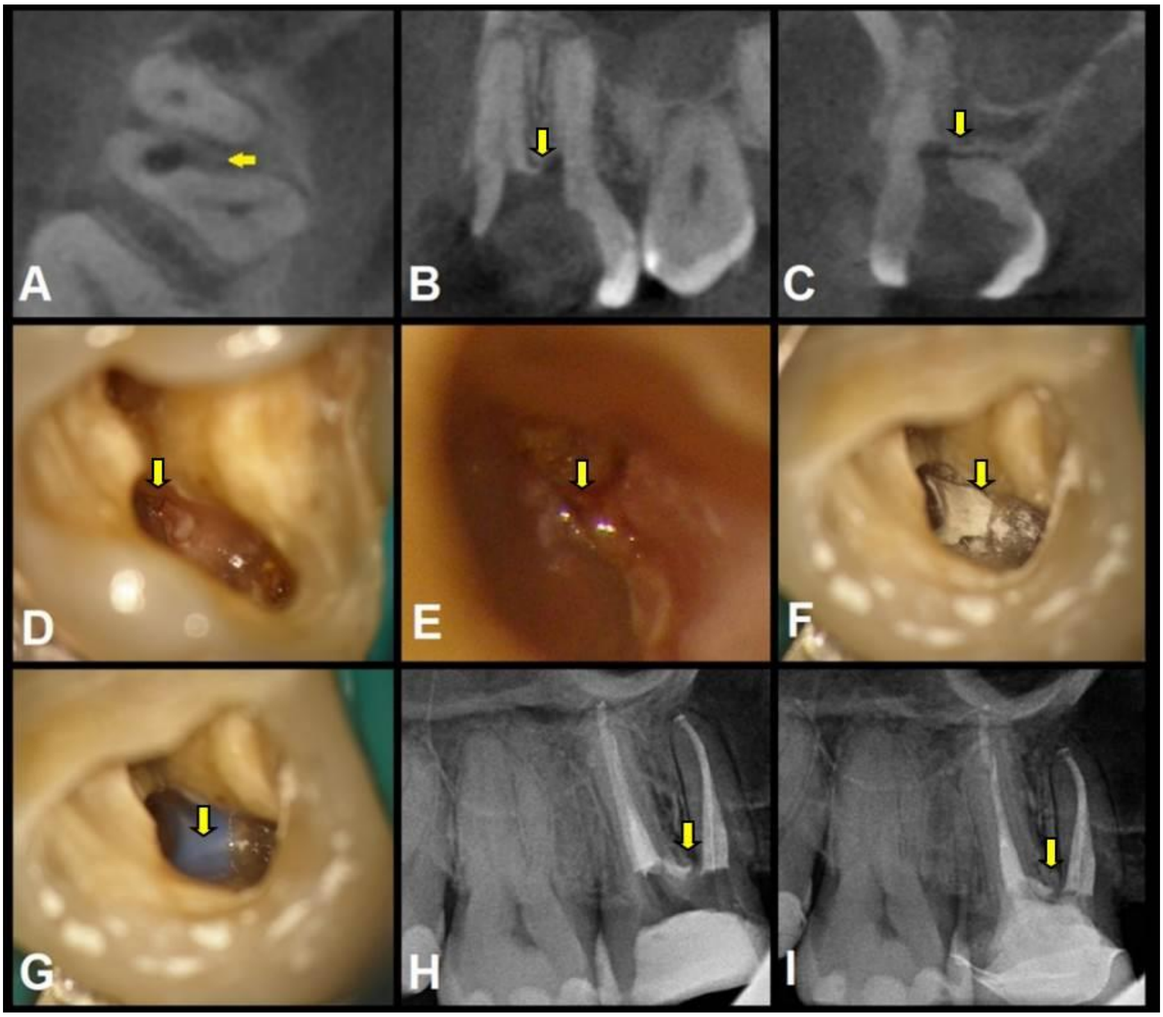

Figure S1. The site of clinical use of mineral trioxide aggregate (MTA). (A) The preoperative CBCT of tooth 27 was suggestive of a perforation (arrow) – an axial plane; (B) The frontal plane; (C) The sagittal plane; (D) The clinical photograph of the pulp chamber floor immediately prior to perforation repair; (E) The magnification of perforation site; (F) The perforation repaired with MTA Repair HP; (G) Calcium silicate cement covered with a flowable composite (blue); (H) The final periapical radiograph showing correctly repaired perforation and well-obturated root canals; (I) The 2-year follow-up; the periapical radiograph shows a healthy crowned molar in full function. The tissue architecture appears to be normal in the perforation site; no evidence of peridontitis.

Consent for the case presented in this paper was given by the Bioethics Committee of the Pomeranian Medical University in Szczecin no KB-0012/36/06/2020/Z. A 44-year-old man was referred in 2018 for evaluation and possible treatment of tooth 27. Patient had no significant medical history. The tooth was non-tender to pressure and percussion. Clinical examination and CBCT suggested a perforation of the pulp chamber floor (A-F). A non-surgical repair of the perforation with calcium silicate cement was chosen. A rubber dam was used for isolation, the perforation site was irrigated with 1% sodium hypochlorite to control haemorrhage and allow visualization of the perforation. Gutta-percha cones were placed in the root canal orifices in order to prevent any reparative material dislodging into canal, and the perforation was repaired with MTA Repair HP (Angelus, Londrina, Brazil) (F). Then, the calcium silicate cement was covered with a flowable composite (G) and conventional root canal treatment was performed. The root canals were finally filled with AH Plus and thermoplasticized gutta-percha (H). Immediately following the procedure, the patient reported no symptoms. Four weeks later, the tooth was restored using FRC post and a prosthetic crown. The 24-month follow-up revealed a normal image for the surrounding tissues and the patient remained asymptomatic (I).

1S. MMP-2 and MMP9 activity in macrophages measurement by zymography method

The samples (25µg) were mixed with Laemmli Sample Buffer (Bio-Rad, Germany) and loaded into wells. The proteins were separated in 7.5% polyacrylamide gels containing 1 mg/ml gelatin and sodium dodecyl sulfate (SDS). After electrophoresis, the gels were washed with renaturing buffer (2.5% Triton™ X-100, Sigma-Aldrich, Germany) and incubated in developing buffer (50 mM Tris, 10 mM CaCl2, 0.02% NaN3, pH=7.4, Sigma-Aldrich, Germany) overnight in 37°C. The gels were washed with water and incubated for 1h in Coomassie Brilliant Blue R-250 (Thermo Fisher Scientific, USA) staining solution. The staining buffer was removed and the gels were incubated with destaining solution (10% methanol, 5% acetic acid, Merck, Germany) until areas of proteolytic activity were clearly visible. Protease bands were detected by absence of Coomassie Brilliant Blue R-250 staining of digested gelatin. Normal serum was used as a control. The bands were qualitatively and quantitatively analyzed with Image Lab Software 6.1.0 (Bio-Rad Laboratories, Inc.).

A.

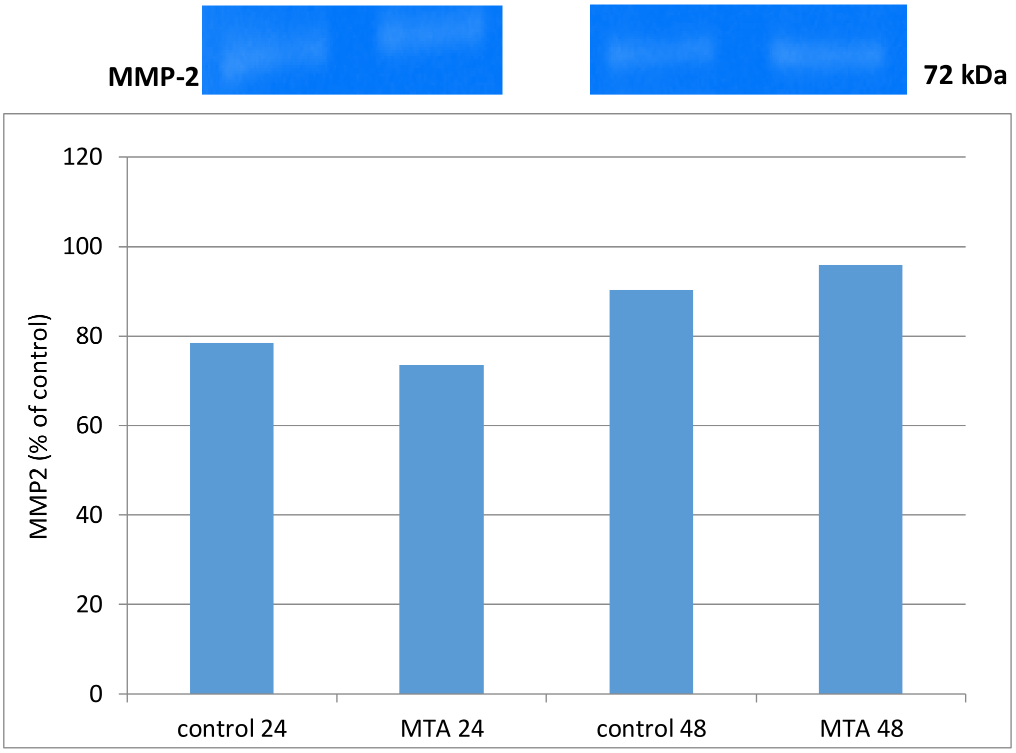

B.

Figure S2. MMP-2 metalloproteinase activity in monocytes (A) equal protein amounts (25 µg) of protein was prepared for gelatin zymography analysis (B) The densitometry values of the proteolytic bands corresponding to MMP-2 is presented as pixel intensity × mm^2 and were normalized to actin to correct for loading. Cells were cultured with mineral trioxide aggregate (MTA Repair HP) for 24 h (MTA24) and 48 h (MTA48) in RPMI medium with 10% FBS. Following incubation, the cells were scraped and protein concentration was measured. The control cells were incubated in 10% FBS RPMI medium for 24h –

control 24 (C24) or for 48h – control 48 (C48). The experiments were conducted as three separate assays. There was no statistically significant differences vs apropriate control group.

Monocytes incubated in the presence of MTA Repair HP for 24 and 48 h (MTA24 vs MTA48) did not show statistically significant changes in activity of the analysed enzyme ($p \geq 0.05$, Mann Whitney U test).

A.

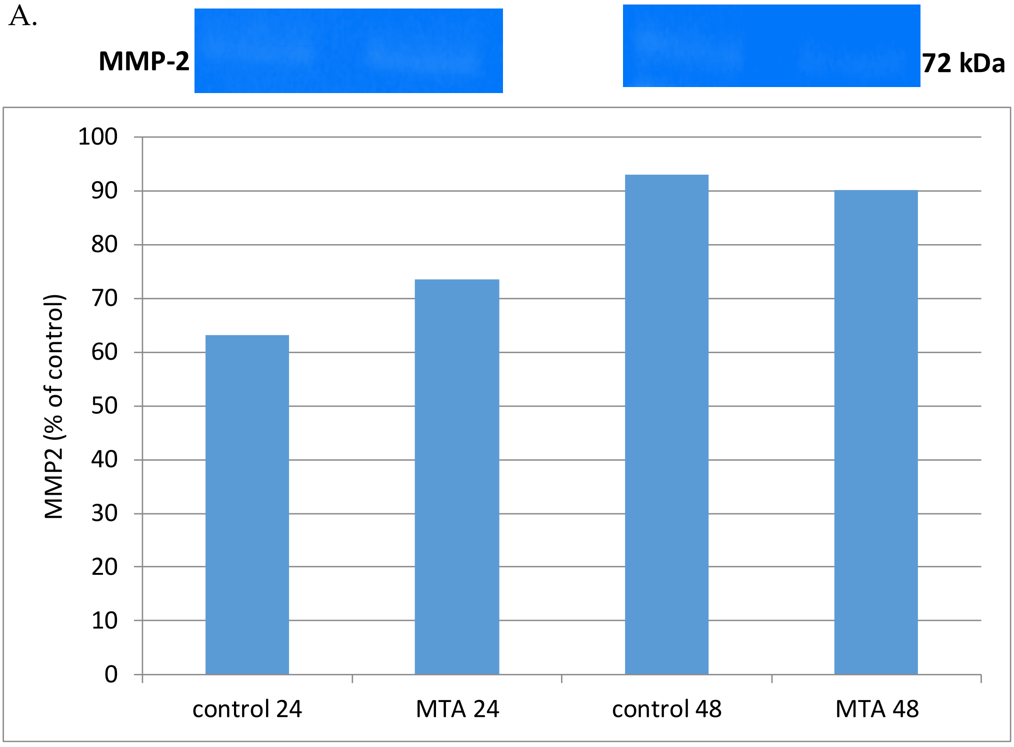

Figure S3. MMP-2 metalloproteinase activity in macrophages (A) equal protein amounts (25 µg) of protein was prepared for gelatin zymography analysis (B) The densitometry values of the proteolytic bands corresponding to MMP-2 is presented as pixel intensity × mm^2 and were normalized to actin to correct for loading. Cells were cultured with mineral trioxide aggregate (MTA Repair HP) for 24 h (MTA24) and 48 h (MTA48) in RPMI medium with 10% FBS. Following incubation, the cells were scraped and protein concentration was measured. The control cells were incubated in 10% FBS RPMI medium for 24h – control 24 (C24) or for 48h – control 48 (C48). The experiments were conducted as three separate assays. There was no statistically significant differences vs appropriate control group.

Macrophages incubated in the presence of MTA Repair HP for 24 and 48 h (MTA24 vs MTA48) did not show statistically significant changes in activity of the analysed enzyme ($p \geq 0.05$, Mann Whitney U test).

A. **MMP-9**

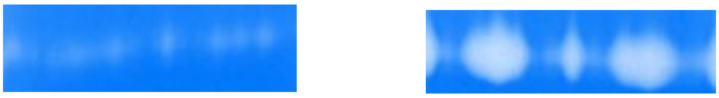

82 kDa

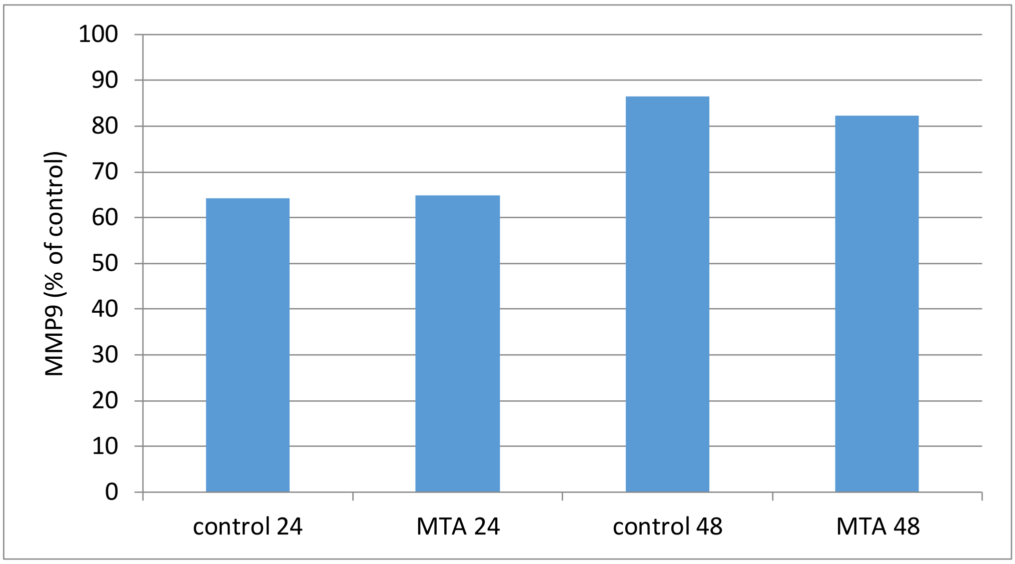

B.

Figure S4. MMP-9 metalloproteinase activity in monocytes (A) equal protein amounts (25 μg) of protein was prepared for gelatin zymography analysis (B) The densitometry values of the proteolytic bands corresponding to MMP-9 is presented as pixel intensity × mm² and were normalized to actin to correct for loading. Cells were cultured with mineral trioxide aggregate (MTA Repair HP) for 24 h (MTA24) and 48 h (MTA48) in RPMI medium with 10% FBS. Following incubation, the cells were scraped and protein concentration was measured. The control cells were incubated in 10% FBS RPMI medium for 24h – control 24 (C24) or for 48h – control 48 (C48). The experiments were conducted as three separate assays. There was no statistically significant differences vs appropriate control group.

A.

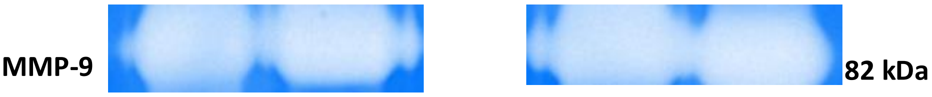

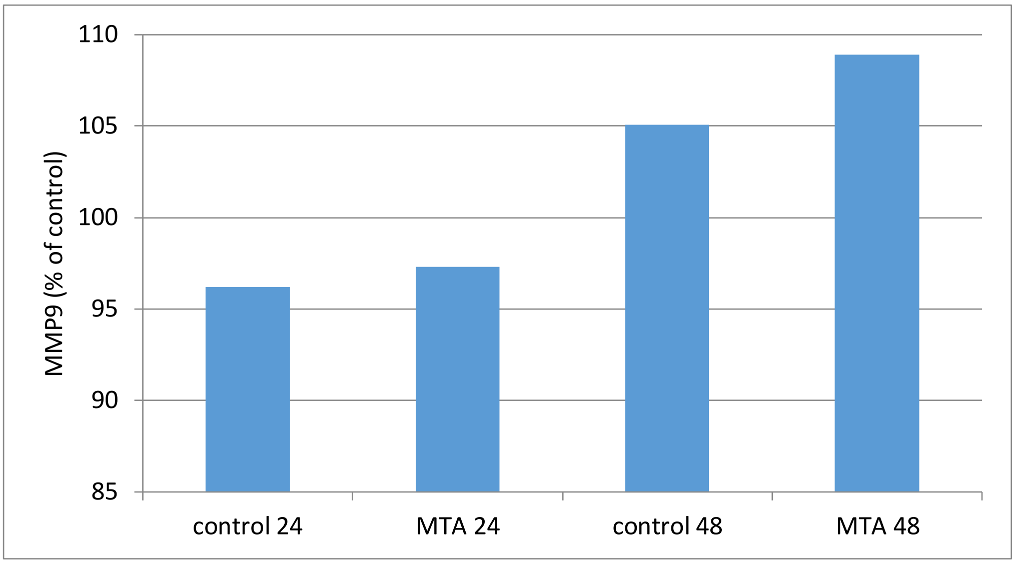

B.

Figure S5. MMP-9 metalloproteinase activity in macrophages (A) equal protein amounts (25 μg) of protein was prepared for gelatin zymography analysis (B) The densitometry values of the proteolytic bands corresponding to MMP-9 is presented as pixel intensity × mm^2 and were normalized to actin to correct for loading. Cells were cultured with mineral trioxide aggregate (MTA Repair HP) for 24 h (MTA24) and 48 h (MTA48) in RPMI medium with 10% FBS. Following incubation, the cells were scraped and protein concentration was measured. The control cells were incubated in 10% FBS RPMI medium for 24h – control 24 (C24) or for 48h – control 48 (C48). The experiments were conducted as three separate assays. There was no statistically significant differences vs appropriate control group.

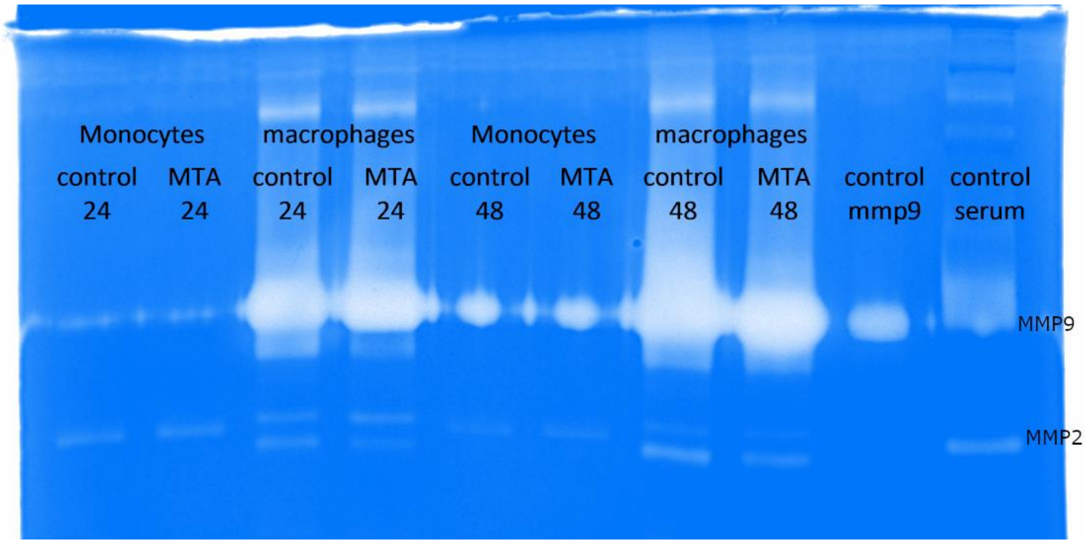

Figure S6. MMP-9 and MMP-2 metalloproteinase activity in monocytes and macrophages – summary zymography analysis. Equal protein amounts (25 µg) of protein was prepared for gelatin zymography analysis. Cells were cultured with mineral trioxide aggregate (MTA Repair HP) for 24 h (MTA24) and 48 h (MTA48) in RPMI medium with 10% FBS. Following incubation, the cells were scraped and protein concentration was measured. The control cells were incubated in 10% FBS RPMI medium for 24h – control 24 (C24) or for 48h – control 48 (C48). The experiments were conducted as three separate assays. There was no statistically significant differences vs appropriate control group.

The results of our study confirm that mineral trioxide aggregate (MTA Repair HP) does not alter MMP-2 and MMP-9 activity in the cultured monocytes/macrophages.

2S. Chemical properties of MTA

Three powder ingredients basically constitute MTA: portland cement (75%), bismuth oxide (20%) and gypsum (5%). The patent specification of MTA states that it contains calcium oxide (50–75 wt %) and silicon oxide (15–20 wt %), which together constitute 70-95% of the cement. When the raw materials are blended, tricalcium silicate, dicalcium silicate, tricalcium aluminate, tetracalciumaluminoferrite are produced [1]. MTA is available in two colours: grey and white MTA, with the major difference being the concentrations of Al_2O_3, MgO and FeO [24]. The traditional composition of MTA was known to show some disadvantages, such as long setting time, tooth and marginal gingiva discolouration or difficult handling [2,3]. The latter disadvantage was frequently reported when performing filling of root-end cavities and furcation or root perforation [4]. However, to counter the negative aspects of MTA application, new formulations have been introduced recently, such as MTA Repair HP (Angelus, Londrina, PR, Brazil) or MTA Vitalcem [2]. The main components of MTA HP powder are tricalcium silicate, dicalcium silicate, tricalcium aluminate, calcium oxide, calcium carbonate (filler material) and calcium tungstate (radiopacifier). The components of the liquid supplied for mixing with the cement powder are water and plasticizing agent. According to the manufacturer, in comparison with White MTA, the new material shows superior properties such as high-plasticity and improved physical properties [1]. MTA Repair HP is based on the conventional MTA formulation, however it contains calcium tungstate as radiopacifier and the mixing liquid with a plasticizer agent. Among the suggested applications of the said material are: root-end filling, pulp capping, pulpotomy, apexogenesis, apexification as well as the repair of root canal perforations. As the manufacturer's instructions state, this new formula maintains the chemical properties of the original MTA, but additionally shows

improved physical properties pertaining to manipulation. The composition of MTA Vitalcem is comparable to that of conventional MTA, however the former contains zirconium dioxide as radiopacifier. The suggested uses of MTA Vitacem are: root-end filling, perforation repair, root resorption, apexification, and pulp capping. Its antimicrobial and regenerative properties are similar to those of the conventional MTA [5].

References

1. Macwan, C.; Anshula Deshpande, A. Mineral trioxide aggregate (MTA) in dentistry: A review of literature. *J. Oral Res. Rev.* **2014**, *6*, 71–74.
2. Silva, E.J.; Carvalho, N.K.; Zanon, M.; Senna, P.M.; De-Deus, G.; Zuolo, M.L.; Zaia, A.A. Push-out bond strength of MTA HP, a new high-plasticity calcium silicate-based cement. *Braz. Oral Res.* **2016**, *30*, doi:10.1590/1807-3107BOR-2016.vol30.0084.
3. Parks, W.C.; Wilson, C.L.; Lopez-Boado, Y.S. Matrix metalloproteinases as modulators of inflammation and innate immunity. *Nat. Rev. Immunol. Engl.* **2004**, *4*, 617–629, doi:10.1038/nri1418.
4. Ferreira, C.M.A.; Sassone, L.M.; Gonçalves, A.S.; de Carvalho, J.J.; Tomás-Catalá, CJ.; García-Bernal, D.; Oñate-Sánchez, R.E.; Rodríguez-Lozano, F.J.; Silva, E.J.N.L. Physicochemical, cytotoxicity and in vivo biocompatibility of a high-plasticity calcium-silicate based material. *Sci. Rep.* **2019**, *9*, 3933, doi:10.1038/s41598-019-40365-4.
5. Abou ElReash, A.; Hamama, H.; Abdo, W.; Wu, Q.; Zaen El-Din, A.; Xiaoli, X. Biocompatibility of new bioactive resin composite versus calcium silicate cements: An animal study. *BMC Oral Health* **2019**, *19*, 194, doi:10.1186/s12903-019-0887-1.
